# Software engineering for reproducible pipeline development in bioinformatics

**DOI:** 10.3389/fbinf.2026.1824590

**Published:** 2026-09-03

**Authors:** Daniel Pérez-Rodríguez, Alba Nogueira-Rodríguez, Jorge Vieira, Cristina P. Vieira, Daniel Glez-Peña, Hugo López-Fernández

**Affiliations:** 1 AA1 Research Group, Universidade de Vigo, Departamento de Química Física, Facultade de Ciencias, Ourense, Spain; 2 SING Research Group, Galicia Sur Health Research Institute (IIS Galicia Sur), Vigo, Spain; 3 Instituto de Investigação e Inovação em Saúde (i3S), Universidade do Porto, Porto, Portugal; 4 Instituto de Biologia Molecular e Celular (IBMC), Porto, Portugal

**Keywords:** bioinformatics, pipeline development, reproducibility, software engineering, workflow management systems (WMSs)

## Abstract

Reproducibility in bioinformatics remains challenging despite the availability of workflow management systems and mature computational infrastructures. This work presents a software-engineering perspective for developing reproducible bioinformatics pipelines, with emphasis on pipeline-specific code. We reinterpret the SOLID principles in this context at two levels: workflow management systems (e.g., Nextflow, Snakemake, and Compi) and pipeline implementation. Our approach promotes pipeline designs based on well-defined task interfaces, explicit input/output specifications, and a clear separation between compute tasks and glue/adaptation tasks, in order to improve flexibility, reuse, and maintainability. The paper provides practical guidance for robust and reproducible pipeline development, including systematic validation checks (environment, inputs, and runtime), standardized project organization, and comprehensive testing strategies using both real and synthetic data within continuous integration workflows. It also discusses how modular ecosystems (such as nf-core modules and Snakemake wrappers) support these principles in community-driven environments. Finally, we relate these recommendations to FAIR-oriented research software guidelines (FAIR4RS and FAIRsoft), showing how core engineering practices strengthen robustness, portability, and long-term sustainability, thereby supporting reproducibility in bioinformatics pipelines.

## 1 Introduction

The growing scale and complexity of biological data analysis has made workflow systems and computational pipelines central to modern bioinformatics (Perkel, 2019). At the same time, reproducibility remains one of the most studied and debated topics in science (Grüning et al., 2018). Despite the availability of guidelines and enabling technologies, achieving reproducible analyses in practice is still challenging, particularly when pipelines combine multiple software components, complex parameterizations, and large evolving datasets.

This situation motivates adopting a software-engineering perspective on pipeline development, which comes with some particularities when compared to software development in other areas. Bioinformatics pipelines are not only scientific artefacts but also long-lived software systems that must satisfy functional requirements (produce correct outputs) and non-functional requirements such as portability, robustness, maintainability, and usability. A central difficulty is that pipeline code typically spans two layers: (i) the workflow management system (WMS), which provides the execution model and orchestration semantics, and (ii) the pipeline implementation itself, which includes task code, glue scripts, and reusable utilities. Pipeline developers usually choose one WMS and then adhere to its constraints and requirements. Although many WMS exist (Perkel, 2019), including Galaxy, CWL (Common Workflow Language), or Cromwell, among others, the most widely adopted in bioinformatics are Nextflow (Di Tommaso et al., 2017) and Snakemake (Köster and Rahmann, 2012). We also include Compi (López-Fernández et al., 2021), a WMS developed by our group, as a complementary running example throughout this article. Improving reproducibility and related software qualities therefore requires practices that can be applied systematically across both layers: disciplined versioning and dependency management, explicit interfaces and contracts between tasks (a discipline examined in detail in section 2), comprehensive testing strategies, and systematic validation through checks at multiple stages of execution.

In this work, we consolidate practical recommendations for engineering reproducible bioinformatics pipelines, focusing on the pipeline-specific code that complements workflow management systems (WMSs). The main contribution is a software-engineering approach that organizes reproducible pipeline development around explicit task contracts (i.e., interfaces), separation of compute and adaptation logic, and a two-level reinterpretation of the SOLID principles across both WMSs and pipeline implementations. This reinterpretation provides a practical design vocabulary for reasoning about interfaces, coupling, extension, substitutability, and separation of concerns in workflow-oriented systems, while helping to improve pipeline flexibility, maintainability, and reusability.

To develop this approach, we first revisit the SOLID principles in the context of pipeline development and discuss how interface-oriented task design helps establish clear contracts between components. We then review how common WMS ecosystems support modular libraries and reusable components. Finally, we connect these design principles with concrete, actionable recommendations covering four critical areas: (i) robustness through systematic validation checks at environment, input, and execution stages; (ii) project organization conventions, including directory layouts and testing infrastructure; (iii) reproducibility practices encompassing version control, containerization, and comprehensive testing with both real and synthetic data; and (iv) strategies for designing reusable and adaptable pipeline components. We also briefly relate these recommendations to FAIR-oriented guidance for research software, including FAIR4RS (Barker et al., 2022) and FAIRsoft (Martín del et al., 2024). This approach is sufficiently precise that its design rules can be formalised as a reusable skill for AI-assisted development tools, a practice increasingly common in modern software engineering, where established best practices are encoded as executable guides rather than remaining tacit knowledge. To demonstrate this approach, we present a case study in which an existing pipeline is refactored using an AI-assisted workflow guided by a skill that encodes the recommendations proposed in this paper.

## 2 Flexible, reusable and maintainable bioinformatics pipelines code with SOLID design principles

While computational pipelines are often conceived as monolithic scripts or “just-get-it-working” workflows, a software-engineering perspective exposes hidden risks such as fragility, excessive coupling, limited reusability, and difficulty evolving. The SOLID principles, a set of five object-oriented design guidelines formulated by Robert C. Martin (Martin, 2003), provide a useful design vocabulary for addressing these risks. Table 1 summarises each principle in its canonical form.

**TABLE 1:** The five SOLID principles in their canonical formulation.

Principle	Statement
Single responsibility (SRP)	A module should have one and only one reason to change
Open/Closed (OCP)	Software entities should be open for extension, but closed for modification
Liskov substitution (LSP)	Objects of a superclass should be replaceable with objects of a subclass without altering correctness
Interface segregation (ISP)	Clients should not be forced to depend on interfaces they do not use
Dependency inversion (DIP)	Depend on abstractions, not on concretions

These principles were originally conceived in the context of object-oriented software, but their underlying intent, reducing coupling, increasing cohesion, and enabling safe extension, translates naturally to the design of computational pipelines. We examine SOLID at the WMS level not because our audience are WMS developers, but because pipeline developers interact with a WMS every day. Recognising SOLID in a tool they already use and trust, and understanding how these principles account for its flexibility and reusability, provides the conceptual grounding to apply the same discipline at the pipeline level, where the design decisions are entirely in their hands. Ultimately, adherence to these principles is what makes a pipeline flexible in the face of change, reusable across projects and teams, and maintainable over the long arc of a research project. In the following subsections we examine how SOLID manifests at two distinct levels: first, in the design of workflow management systems themselves; and second, in the decisions made by pipeline developers when implementing their own workflows.

### 2.1 SOLID at the workflow management system level

From a software perspective, a computational pipeline is data-processing software composed of tasks that consume input data and produce intermediate or final outputs, with a specific execution order. Some tasks must run sequentially because their outputs feed subsequent tasks, while others can run in parallel when they have no dependencies. The term “pipeline” traditionally denotes a linear sequence of steps; “workflow” describes a more general collection of tasks that may include parallel branches. Tools that help bioinformaticians define and run these processes are known as workflow management systems (WMS) (Perkel, 2019). Regardless of terminology, most WMS model workflows as directed acyclic graphs (DAGs) (Mölder et al., 2021), where nodes represent tasks and directed edges indicate dependencies between them.

A WMS is, in software-engineering terms, a framework: it provides a fixed execution engine and imposes an abstract model of computation that users extend by supplying their own task implementations. At this level of abstraction, the building blocks are minimal: executable units, dependencies between them, and runners that determine how and where those units are executed. File formats, biological parameters, and domain-specific tool chains are entirely invisible to its core. Indeed, although many WMS were originally conceived in the context of bioinformatics, their engines are completely domain-agnostic. As a consequence, several SOLID principles are not merely recommendations when using a WMS, they are structural properties of the WMS itself.

#### 2.1.1 Single Responsibility Principle

A WMS enforces, by design, the decomposition of an analysis into discrete, named execution units. Each unit has a bounded scope: it encapsulates a single piece of executable code and declares its dependencies on other units. The WMS itself is also internally decomposed, as scheduling, dependency resolution, logging, and execution are handled by separate subsystems, which is precisely what SRP prescribes. Beyond its own internal organisation, the WMS actively encourages users to adopt the same discipline: a more fine-grained task decomposition improves parallelisation, simplifies failure recovery, and makes individual steps independently reusable. The resulting pipeline is also easier to maintain, because any future change, whether to a tool version, an output format, or a parameter, affects only the single task responsible for it, leaving the rest of the workflow untouched.

#### 2.1.2 Open/closed principle

The execution engine of a WMS is a stable, closed component: users do not modify it to accommodate new analyses. Instead, they extend the system by implementing new tasks that conform to the framework’s interface contract. This is the defining characteristic of OCP. Some WMS take this further by allowing the execution mechanism itself to be extended without modifying task definitions. Compi (López-Fernández et al., 2021), for instance, supports *custom runners*, pluggable components that determine how and where a task is executed (locally, via a container, on a SLURM cluster, or on a cloud provider), while leaving the task definitions untouched. Nextflow achieves a similar separation through its configurable executor abstraction (Di Tommaso et al., 2017). In both cases the system is open for extension at the execution layer while the analytical logic remains closed for modification.

A third category of extension point, alongside tasks and runners, is the provision of *observability hooks*: interfaces that allow external components to react to execution events (task start, task completion, failure, workflow end) without any modification to the engine itself. Nextflow exposes this through its trace observer plugin system: a conforming plugin is deployed entirely outside the engine and outside any pipeline, and the engine invokes it uniformly alongside every workflow it runs. Snakemake offers a narrower equivalent through its logger plugin interface, which supports observation of events without the ability to alter execution (Köster and Rahmann, 2012). In both cases the engine remains closed: the hook is a new, self-contained artefact that conforms to a prescribed interface. The distinction between WMS in this regard is not about whether OCP holds at the engine level, but about how rich and formally specified the extension point is, which has direct consequences for pipeline developers, as we discuss in the following section. More broadly, the separation between a stable execution core and open extension points is what makes a WMS flexible, because it can be extended with new capabilities without breaking existing pipelines, and maintainable, because new requirements are satisfied by adding code rather than modifying shared infrastructure.

#### 2.1.3 Liskov Substitution principle

For a task to be a valid participant in a WMS-managed workflow, it must satisfy the contract imposed by the framework: declare its dependencies and executable code in the manner the system expects, and report its execution state correctly. Any task that fulfils this contract can be scheduled, monitored, and retried by the engine without special treatment, regardless of what it does internally. The same applies to pluggable extension components such as runners or executors: a custom runner must satisfy the interface that the framework prescribes for runners, so that the engine can invoke and supervise it uniformly. When this principle is violated, the engine behaves unexpectedly not because of a bug in the system itself, but because one of its participants failed to honour the agreed contract. The resulting errors are the responsibility of the implementor, not of the system. This uniform substitutability is what makes a WMS flexible: the execution engine can grow its ecosystem of tasks, runners, and plugins without any risk of the core breaking, because correctness is guaranteed by contract adherence rather than by specific implementation knowledge.

#### 2.1.4 Interface Segregation Principle

A WMS exposes a range of capabilities to the pipeline developer: task execution, custom runners, observability hooks, executor configuration, container integration, and more. ISP, viewed from the perspective of the client, prescribes that a pipeline should not be forced to depend on mechanisms it does not use. Well-designed WMS honour this by making all capabilities beyond the minimal task contract strictly opt-in: a pipeline that needs nothing beyond basic sequential execution declares only tasks and their dependencies, and the existence of runners, hooks, or cloud executors is entirely invisible to it. Only when the developer’s needs grow does the relevant capability need to be understood and adopted. A WMS that instead required every pipeline to explicitly declare a runner, configure an executor, and register an observability hook, even if only to leave them empty, would violate ISP: the pipeline would be forced to depend on interfaces it has no use for. The practical consequence of this segregation is a genuinely incremental learning curve: a bioinformatician can write a functional pipeline on day one with minimal knowledge of the framework, and progressively adopt richer capabilities as their use case demands, without the earlier, simpler pipelines ever needing to change. This opt-in architecture also makes pipelines more flexible, because they can adopt new capabilities without carrying accidental dependencies on mechanisms they do not use, and more maintainable, because each pipeline is coupled only to the subset of the WMS it actually needs.

#### 2.1.5 Dependency Inversion principle

The execution engine of a WMS does not depend on the concrete implementations of individual tasks, but on the minimal abstract interface that the framework requires from any task: a piece of executable code and a declaration of its dependencies with respect to other tasks. The engine schedules, monitors, and retries tasks based solely on this abstraction, without any knowledge of what the task does, which tools it invokes, or what data it manipulates. Crucially, this reflects a true inversion of dependencies: it is not the framework that depends on the pipeline, but the pipeline that depends on the framework. Nextflow, Snakemake, or Compi do not know about, nor are modified by, the thousands of pipelines built on top of them. It is the pipeline developer who must conform to the framework’s standards, not the other way around. This asymmetry is precisely what makes a WMS a reusable, stable piece of infrastructure. The inversion is further reinforced in WMS that decouple the execution environment from the task definition: the framework depends on an abstract notion of a runner or executor, while concrete implementations, Docker, Singularity, SLURM, AWS Batch, are injected via configuration. The analytical core thus remains portable and environment-agnostic. Because the engine depends only on abstractions, it is also flexible: no concrete pipeline, tool, or execution environment can destabilise the core, and the WMS remains reusable across entirely different scientific domains without any modification.

### 2.2 SOLID at the pipeline level

At the WMS level, the domain is deliberately absent from the framework’s concerns. Descending to the pipeline level means reintroducing that domain: file formats, tool parameters, biological data contracts, and the specific analytical logic that gives the workflow its scientific meaning. We propose to also follow the SOLID guidelines at this level, as a means to produce pipeline code that is flexible in the face of changes in tools or data formats, genuinely reusable across projects, and maintainable by collaborators who were not present at the time of its creation.

Beyond the structural guarantees that a WMS provides, the quality of a pipeline depends heavily on the design decisions made by its developer. Applying SOLID principles at this level requires first establishing a precise model of what a task is and how it should be composed.

Unlike object-oriented languages, where interfaces are first-class constructs enforced by the compiler, WMS do not provide a mechanism to formally declare the domain-level interface of a task. The framework knows about code and dependencies; it knows nothing about whether a task expects a sorted BAM or a raw FASTQ. This contract lives only in the developer’s design intent. Rather than seeing this as a limitation, we encourage pipeline developers to embrace it as a design discipline: before writing a single line of code, define each task as a black box with explicit inputs, outputs, and parameters. Ask: *what would this task look like if I reimplemented it with a different tool, or in a different language?* If that question has a clear answer, the interface is well-defined. In WMS such as Compi, this discipline has a direct practical payoff: a task’s implementation can be swapped simply by pointing its src attribute to a different script, whether written in Bash, Python, or R, or invoking an entirely different bioinformatics tool, without modifying the pipeline structure at all.

**FIGURE 1:**
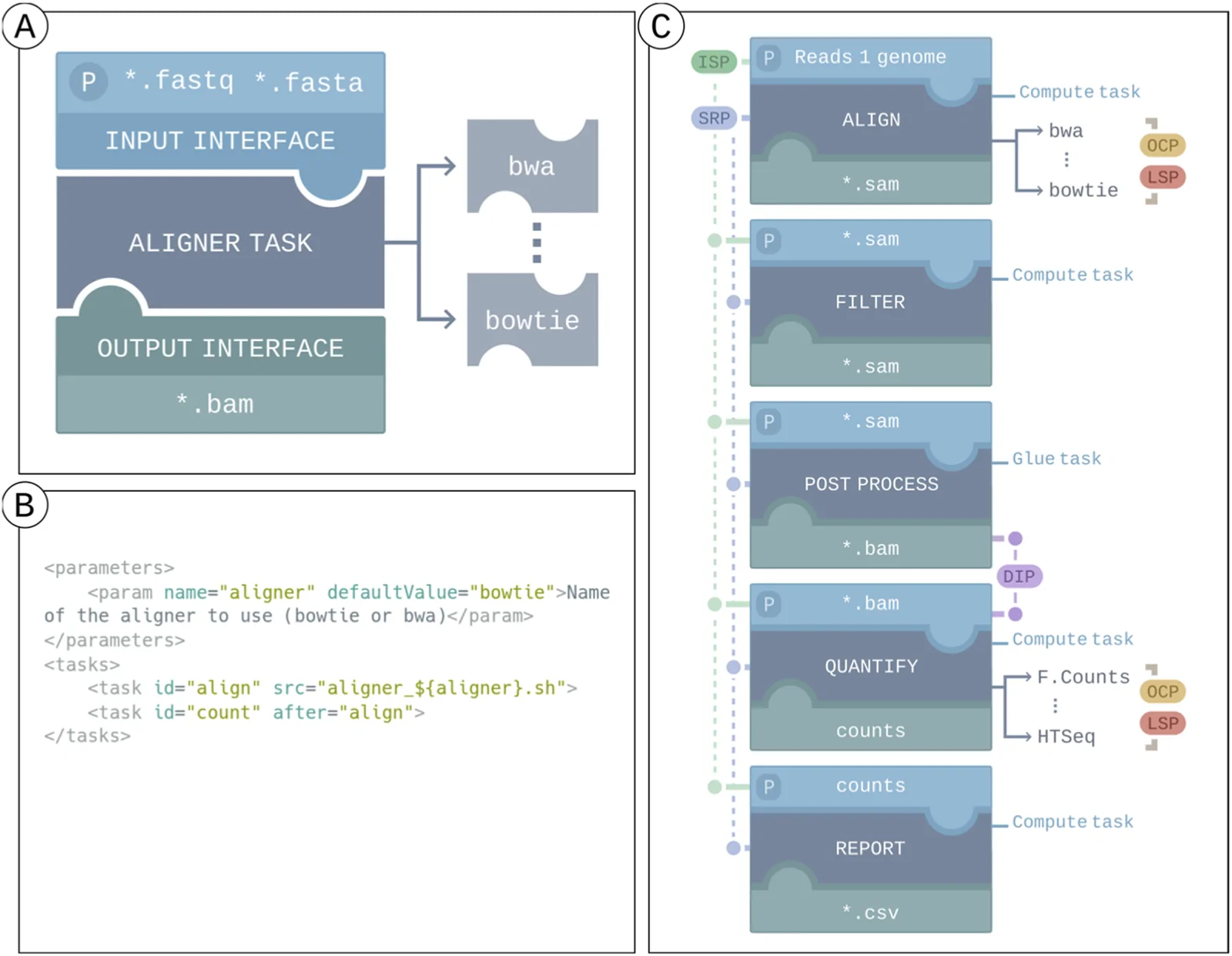
Panel **(A)** The three elements of a task: input and output interfaces and parameters. Panel **(B)** Defining interfaces reduces tool switching to a simple value change, as illustrated in this Compi pipeline snippet. Panel **(C)** Example of a SOLID-compliant pipeline architecture. Each pipeline stage is modeled as an independent task that explicitly defines its input and output interfaces, enabling interchangeable implementations while preserving workflow compatibility. The architecture illustrates the application of SOLID principles—represented by the labeled ovals—across the pipeline, where compute tasks (e.g., alignment and quantification) may be implemented by multiple tools (e.g., bwa, bowtie, FeatureCounts, or HTSeq) without affecting downstream stages. Intermediate outputs are connected through well-defined contracts, promoting modularity, extensibility, maintainability, and tool substitution.

As depicted in Figure 1 (panel A), a task is fundamentally characterised by three elements: an *input interface* (the files and formats it consumes), an *output interface* (the files and formats it produces), and a set of *parameters* that govern its behaviour. Parameters fall into two categories: task-level parameters, which control logical decisions internal to the task (e.g., filter_quality = true, min_coverage = 10), and tool-level parameters, which are forwarded verbatim to the underlying software (e.g., bowtie_args = “--very-sensitive” threads = 8). Having this contract clear before writing any code is precisely the exercise we propose: think of the task as a black box, and ask whether a different implementation could satisfy the same contract. If that is possible, then the interface is well-defined. For example, the interface of an align_reads task could be defined as follows:


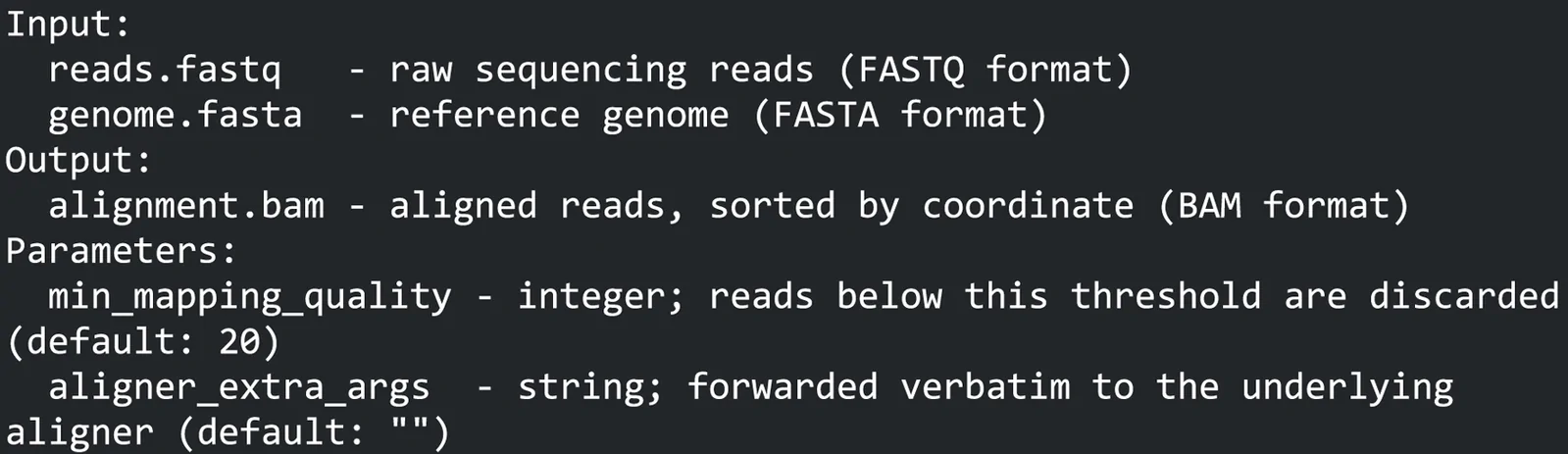


Any implementation that honours these inputs, outputs, and parameter semantics, whether backed by Bowtie2, BWA-MEM2, or any future aligner, is a valid substitute for this task (see example in Figure 1, panel B), as it will be explained below.

This interface-based view supports a useful distinction between two functional roles. *Compute tasks* directly invoke third-party bioinformatics tools and are primarily responsible for executing the tool with appropriate parameters and managing its I/O (e.g., align_with_bowtie, quantify_with_featurecounts). *Glue tasks* (or *adapter tasks*) perform pre- or post-processing, format conversions, metadata transformations, or the orchestration of multiple tools (e.g., convert_sam_to_bam, merge_counts, filter_low_quality_reads). Keeping these two roles separate is essential: embedding glue logic inside compute tasks creates hidden complexity, reduces testability, and couples unrelated concerns. Compute tasks with clean, well-defined interfaces are also the natural candidates for publication as reusable modules in shared libraries, such as nf-core modules or Snakemake wrappers, as discussed in Section 3; glue tasks, being inherently pipeline-specific, rarely make sense outside the context in which they were written, although in some cases they may evolve into a new reusable module that is released out of the context of a pipeline.

By modelling tasks through explicit input/output interfaces and parameter contracts, the pipeline developer establishes the foundation for *interface-based substitutability*, the ability to swap, extend, and compose tasks based on their contracts rather than their internal implementations. The following subsections examine how each SOLID principle guides these design decisions in practice.

#### 2.2.1 Single Responsibility Principle

A task should have one and only one reason to change. In practice, this means that a task’s scope must be bounded by a single, clearly named responsibility: if a task needs to be modified both because the alignment tool changed its command-line interface and because the output naming convention was updated, it has two responsibilities and should be split. Glue code, pre-processing, output renaming, metadata conversion, should not be embedded inside compute tasks but extracted into dedicated tasks and treated as first-class pipeline components. This isolation makes the impact of any change local and predictable: when a file-naming convention changes, only the dedicated renaming task requires modification, leaving the alignment logic untouched. For example, a monolithic align_and_count task conflates at least four distinct reasons to change; decomposing it into align_reads, postprocess_alignment, quantify_counts, and summarise_results ensures that each step changes only when its own responsibility changes, and for no other reason (Figure 1, panel C). The result is a pipeline that is easier to maintain, because the scope of every modification is narrow and well-understood, and more reusable, because each focused task can be extracted and incorporated into a different pipeline without carrying unwanted logic.

#### 2.2.2 Open/closed principle

A well-designed pipeline should be extendable without requiring structural modifications to its core. A common anti-pattern is embedding all task implementation code directly in the pipeline definition file: any change to a task’s logic, however minor, forces the developer to edit the same file that defines the overall workflow structure, mixing two concerns that should remain independent. A cleaner approach is to separate the pipeline structure, the DAG, the task declarations, and the dependency graph, from the concrete implementation of each task. In Compi, this is achieved through the src attribute, which points each task to an external script file; the pipeline definition file describes *what* must be done and *in what order*, while the scripts describe *how* each step is carried out. Snakemake follows the same principle through its script: directive, which delegates a rule’s execution to an external file (Python, R, Bash, or others) while the Snakefile retains only the structural definition.

With this separation in place, *extending* the pipeline has a concrete and precise meaning: suppose the pipeline contains an align_reads task currently implemented with Bowtie2, and the developer wishes to support BWA-MEM2 as an alternative aligner. Extension means creating a new file align_reads_bwamem2.sh, implementing the same interface contract (same expected inputs, same output formats), and updating the src attribute of the task to point to the new file (see example in Figure 1, panel B). Every downstream task remains untouched, and the pipeline structure is unchanged. The old implementation is not modified or deleted; it can coexist alongside the new one. This is what OCP means in practice: growth through addition, not modification.

Taking this further, if the WMS or the pipeline allowed the src reference to be supplied as an external configuration parameter at runtime, extension would not even require touching the pipeline file at all: the developer would simply pass the path to the new implementation when invoking the pipeline. This is not currently supported by all WMS, but it represents the natural direction to which OCP points, and is worth considering as a design goal both for pipeline developers and for WMS authors. The pipeline core thus remains closed for modification and open for extension, provided that the interface each task is expected to fulfil, its inputs, outputs, and parameter contracts, is clearly documented; which, as discussed under LSP, is a discipline the developer must maintain explicitly.

The same logic applies to the other extension points that a WMS exposes at the pipeline level. In Compi, runners are by design self-contained files: adding a new execution environment, a SLURM profile, a Docker wrapper, a cloud provider, always means creating a new runner file, and the pipeline definition is never touched. OCP holds here almost automatically, because the WMS architecture enforces it. Observability hooks follow the same pattern when the WMS supports them cleanly: in Nextflow, adding post-execution monitoring (metrics aggregation, Slack notifications, audit logging) means writing a new plugin that implements the trace observer interface and deploying it externally; the pipeline source is untouched. Snakemake’s onsuccess and onerror directives serve a similar purpose but require adding code inside the Snakefile itself, which means that extending post-execution behaviour comes at the cost of modifying the pipeline. The degree to which OCP holds at the pipeline level therefore depends partly on the extension points the WMS provides, and partly on the discipline the developer applies when structuring task implementations. Where OCP is consistently applied, the pipeline remains maintainable because the structural core is never touched to accommodate new requirements, and stable because existing, tested implementations are not put at risk by extensions.

#### 2.2.3 Liskov Substitution Principle

When two tasks fulfil the same analytical role in a pipeline, they should be interchangeable without altering the correctness of the workflow. This requires that their input/output interfaces, naming conventions, and parameter contracts be mutually consistent. A pipeline that expects a quantification task consuming a BAM file and producing a counts table should operate correctly regardless of whether that task is backed by featureCounts or HTSeq-count, provided both honour the same interface. This substitutability is only possible if the pipeline depends on the interface contract, not on the specific implementation: a new quantification tool (quantify_with_salmon, quantify_with_kallisto) can replace an existing one simply by matching the interface expected by downstream tasks, without touching the rest of the workflow. This situation is illustrated in Figure 1, panel C, by the align and quantify tasks.

However, as established earlier, task interfaces in a pipeline are not formal artefacts enforced by the WMS; they exist only in the developer’s design intent. This makes explicit documentation of each task’s contract a prerequisite for substitutability: if the expected input formats, output naming conventions, and parameter semantics are not written down, different implementations will inevitably diverge, and substitution will silently break the pipeline. LSP at the pipeline level is therefore as much a documentation discipline as a design one. When honoured, it makes the pipeline genuinely reusable: well-documented, interchangeable tasks can be shared, repurposed in new workflows, and published as community modules. It also makes the pipeline robust, because the correctness of the workflow does not depend on the identity of any particular implementation, only on the contract that implementations are required to satisfy.

#### 2.2.4 Interface Segregation Principle

Tasks should depend only on the inputs, outputs, and parameters they actually require. This principle manifests at two levels in a pipeline. At the configuration level, a common anti-pattern is the use of a single monolithic configuration object passed to every task: each task then has access to, and implicitly depends on, configuration keys that are entirely irrelevant to it. A better design partitions configuration into per-task or per-module sections (e.g., config[“alignment”], config[“quantification”]) so that each task reads only its own slice, limiting the blast radius of configuration changes and simplifying testing. At the data level, a task should not be forced to receive a file it only partially uses. For example, a task that extracts gene-level counts from a GTF annotation should not receive the entire genome FASTA alongside it simply because both are available at that pipeline stage; it should declare only the GTF as its input. Forcing unnecessary data dependencies increases coupling, complicates reuse, and makes it harder to reason about what a task actually needs to run correctly. A pipeline that applies ISP consistently is more maintainable because each task’s dependencies are minimal and clearly declared, reducing the cognitive overhead of understanding or modifying any individual step. It is also more robust against configuration drift: a task that reads only its own configuration slice cannot be inadvertently broken by changes to configuration sections it does not own.

#### 2.2.5 Dependency Inversion Principle

When each task imposes its own interface requirements on upstream dependencies, incompatibilities between otherwise independent components become visible and explicit. For instance, if task A produces an unsorted SAM file and task B requires a sorted BAM, that mismatch cannot be silently absorbed: it must be bridged by a dedicated glue task (convert_and_sort_sam_to_bam) that acts as an adapter between the two contracts (this is illustrated in Figure 1, panel C, by the post process task). This asymmetry is precisely what DIP prescribes: the compute task defines the interface it exposes, and it is the glue task that depends on that interface to perform its adaptation work. The compute task does not change to accommodate downstream consumers; instead, glue tasks absorb the coupling. As a consequence, compute tasks become genuinely reusable across different pipelines: featureCounts produces a counts table with a well-defined format regardless of what precedes or follows it; if a different pipeline connects it to incompatible upstream or downstream steps, a new glue task handles the mismatch without touching featureCounts at all. These glue tasks are inherently more pipeline-specific than compute tasks, but making them explicit and first-class is preferable to hiding the adaptation logic inside one of the tasks and blurring its responsibility. Execution environment concerns, container images, Conda environments, resource directives, cluster profiles, should be decoupled from the analytical logic and managed via external configuration, so that the pipeline core remains portable across infrastructures. Taken together, these practices make the pipeline flexible, because compute tasks are insulated from changes in their execution context; reusable, because tasks defined against stable abstractions can be composed into new workflows without modification; and maintainable, because coupling is made explicit and localised in dedicated glue tasks rather than scattered across the analytical logic.

### 2.3 Principles in practice

The five principles described above can be difficult to evaluate in isolation. A concrete design comparison makes their combined effect visible and provides a reference point for the validation and good practices discussed in the sections that follow.

#### 2.3.1 Monolithic vs. modular task design

Consider a pipeline fragment for RNA-seq analysis in which a single task is responsible for the entire analytical chain. This task receives raw reads, a reference genome, and an annotation file; indexes the reference; performs alignment; sorts and filters the resulting alignment; quantifies feature counts; renames outputs according to a pipeline-specific convention; and writes a summary report.


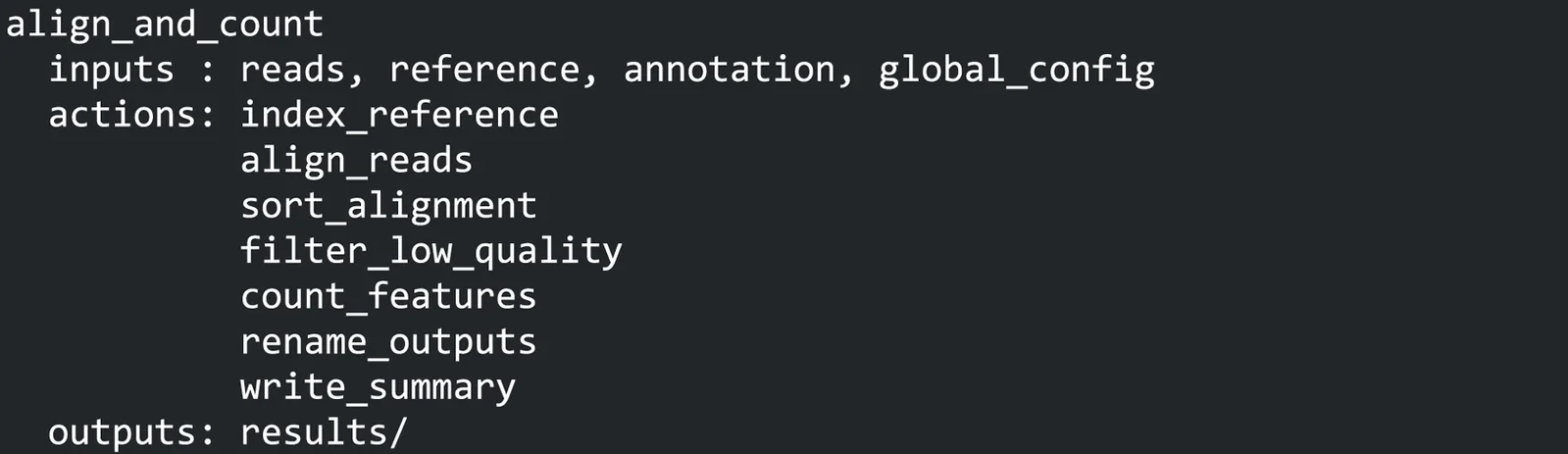


This design concentrates too many responsibilities in a single unit. Any change—substituting the aligner, adjusting the filtering threshold, modifying the output naming scheme, or updating the summary format—requires editing the same task. Testing is only feasible end-to-end, so a failure anywhere inside the task is difficult to localise. The task cannot be reused in a different pipeline without carrying all its embedded assumptions with it. Because all steps share access to a single global configuration block, downstream steps implicitly depend on parameters that are irrelevant to them.

Panel C in Figure 1 presents a simplified representation of a better design, decomposing the work into tasks with bounded, non-overlapping responsibilities. Each task now has exactly one reason to change. Substituting the aligner means replacing only the ALIGN implementation behind a stable contract; no downstream task needs to be modified. Glue tasks handle format or naming mismatches explicitly rather than hiding them inside an analytical step. Each task can be tested independently with a small synthetic input, and failures are localised to the step where they occur. Configuration is segmented so that each task reads only the parameters it requires.

#### 2.3.2 SOLID principles summary

SOLID was originally formulated for object-oriented software design, whereas workflow definitions and pipeline tasks are not object-oriented entities in a strict formal sense. However, it has been shown before how tasks can be specified by well-defined input and output interfaces and, in this context, SOLID terminology is adopted as a design vocabulary for reasoning about such interfaces, coupling, extension points, substitutability, and separation of concerns in workflow-oriented systems. Table 2 summarises how each SOLID principle translates to pipeline design, together with its characteristic anti-pattern and the corresponding recommended design move.

**TABLE 2:** Summary of the five SOLID principles in the context of pipeline design.

Principle	Pipeline interpretation	Common anti-pattern	Recommended design move
SRP	One task, one reason to change	Monolithic multi-purpose tasks	Split compute and glue logic into separate tasks
OCP	Extend by adding implementations, not by rewriting pipeline structure	Editing the pipeline core whenever a tool changes	Swap external task implementations behind stable contracts
LSP	Alternative implementations must remain interchangeable	Two equivalent tasks produce incompatible output conventions	Preserve contracts and output naming semantics across implementations
ISP	Tasks depend only on the configuration and data they actually use	One global configuration block shared by all tasks	Per-task or per-module configuration slices
DIP	Bridge mismatches through explicit adapter tasks rather than modifying compute tasks	Modifying a compute task to satisfy every downstream consumer	Insert dedicated glue tasks that adapt formats and contracts

## 3 Reusable components and modular ecosystems

Focusing on code writing, the most widely used WMS, namely, Nextflow (Di Tommaso et al., 2017) and Snakemake (Köster and Rahmann, 2012), support the creation of modular designs by encouraging the creation of small, single-purpose processes, tasks or rules, and provide different mechanisms to support code reuse and ease maintenance.

Nextflow pipelines are made up of processes connected via the so-called *dataflow* channels. Such processes are idempotent tasks (identified by a hash of its inputs and code) that run in isolated work directories. These definitions impose an interface and allow refactoring and reusing them across different workflows, a property that corresponds to the Liskov Substitution and Dependency Inversion principles discussed in Section 2. The nf-core framework was initially created as a way to provide a curated collection of pipelines implemented according to best-practices and reusable models. It evolved towards a repository of reusable modules and subworkflows that can be reused to build new pipelines (Langer et al., 2025). An nf-core module provides a Nextflow process definition for a specific functional task, developed in compliance with the nf-core standards that include, among others, the declaration of the required software dependencies (using container images and/or Conda packages). The nf-core best practices ensure that contributions, in form of modules, subworkflows and workflows, follow the FAIR principles and proper documentation practices, as well as adhere to the Nextflow community code conventions.

Snakemake workflows are made up of rules that describe how to obtain a set of output files from a set of input files, based on how the Make building system works. This transformation can be specified in a shell command, a block of Python code, an external script (e.g., R or Python), a Jupyter notebook or by a tool wrapper. Beyond such individual rules, Snakemake supports multiple levels of modularization (Mölder et al., 2025). Workflows can be split across Snakefiles using include statements, grouping related rules to improve readability and allow users to focus on specific analysis stages. For larger-scale reuse, workflow modules enable integrating, adapting, and extending external workflows transparently, building complex pipelines from tested components.

Step-wise modularization distinguishes between custom steps (i.e., *glue-code*, analogous to the *glue tasks* described in Section 2), implemented as external scripts or Jupyter notebooks, and recurring tasks, which can use reusable tool wrappers. These wrappers, hosted in a central repository, package both the execution logic and required software environment, therefore ensuring reproducibility. The repository also provides meta-wrappers (or predefined subworkflows) that combine multiple steps, allowing rapid, maintainable pipeline construction from standardized, reusable building blocks.

Both Nextflow and Snakemake emphasize testing and reproducibility as part of modular pipeline development. All nf-core modules and pipelines undergo automated continuous integration to ensure correct execution across environments, while Snakemake wrappers and subworkflows are automatically tested before inclusion in the central repository. Combined with standardized software environments, these practices help modular components remain robust, reusable, and reproducible.

Similarly, in our Compi WMS, pipelines are made up of tasks that run an arbitrary piece of code in any programming language (López-Fernández et al., 2021). Compi does not impose further restrictions and handles parameter visibility according to the task settings. As discussed in Section 2, tasks can indicate a source executable script via the src attribute, decoupling pipeline specification from task code. This way, tasks can be seen as custom modules that are then assembled to build the pipeline and can be easily reused across projects. To do so, pipeline developers must be careful when designing the task interfaces (i.e., input parameters and input/output data formats). Future versions of Compi will include a modular system that allows developers to include predefined modules (i.e., tasks, parameters, runners, and so on) into their developments, further enhancing reusability.

The separation of workflow definition from execution in these WMS, as discussed in Section 2, also enables seamless deployment across diverse computing environments, a key aspect towards truly reproducible pipelines. In Snakemake, each rule can specify its required software stack via Conda environments or container images, which are automatically deployed at runtime. Conda environments provide isolated, reproducible software installations, while containers allow precise control over system libraries, ensuring portability across operating systems and HPC systems. Snakemake can even automatically generate containerized versions of workflows from defined Conda environments, combining reproducibility with development flexibility. Similarly, Nextflow allows each process to declare its execution environment using Conda, Docker, or Singularity, while the workflow logic itself remains independent of the underlying system. This decoupling of implementation and execution ensures that modular workflows can be reliably run on personal computers, cloud platforms, or HPC clusters without modification, enhancing portability, reproducibility, and maintainability. Compi has built-in support for packaging and distributing pipelines via Docker images. In addition, the runners mechanism implemented in Compi allows pipeline developers to specify a custom environment for the desired tasks. Compi provides several generic runners to manage task execution in Docker containers, Slurm or AWS.

Finally, it is worth noting a recent and very specific case of two modular pipelines called auto-phylo (López-Fernández et al., 2023; López-Fernández et al., 2024) and auto-p2docking (Vieira et al., 2025). They were developed by the Phenotypic Evolution Group at i3S, and are aimed at providing a customizable pipeline in which end-users could assemble their own analysis workflows based on the available modules. Unlike traditional pipelines that have a predefined, fixed structure developed to work on a specific WMS, these are like flexible block-building toys that can adapt to the end-user needs.

Both pipelines provide a set of predefined and standardized modules with well-defined input and output files. For instance, the latest auto-phylo version (v3 as of writing this manuscript) provides more than 50 modules for different tasks related with phylogenetic studies. Then, it provides a simple workflow engine that allows users providing, at runtime, a specific pipeline assembly of such modules. A configuration file can be also specified to customise modules behaviour.

## 4 Reliable, maintainable and reproducible pipelines in practice

**FIGURE 2:**
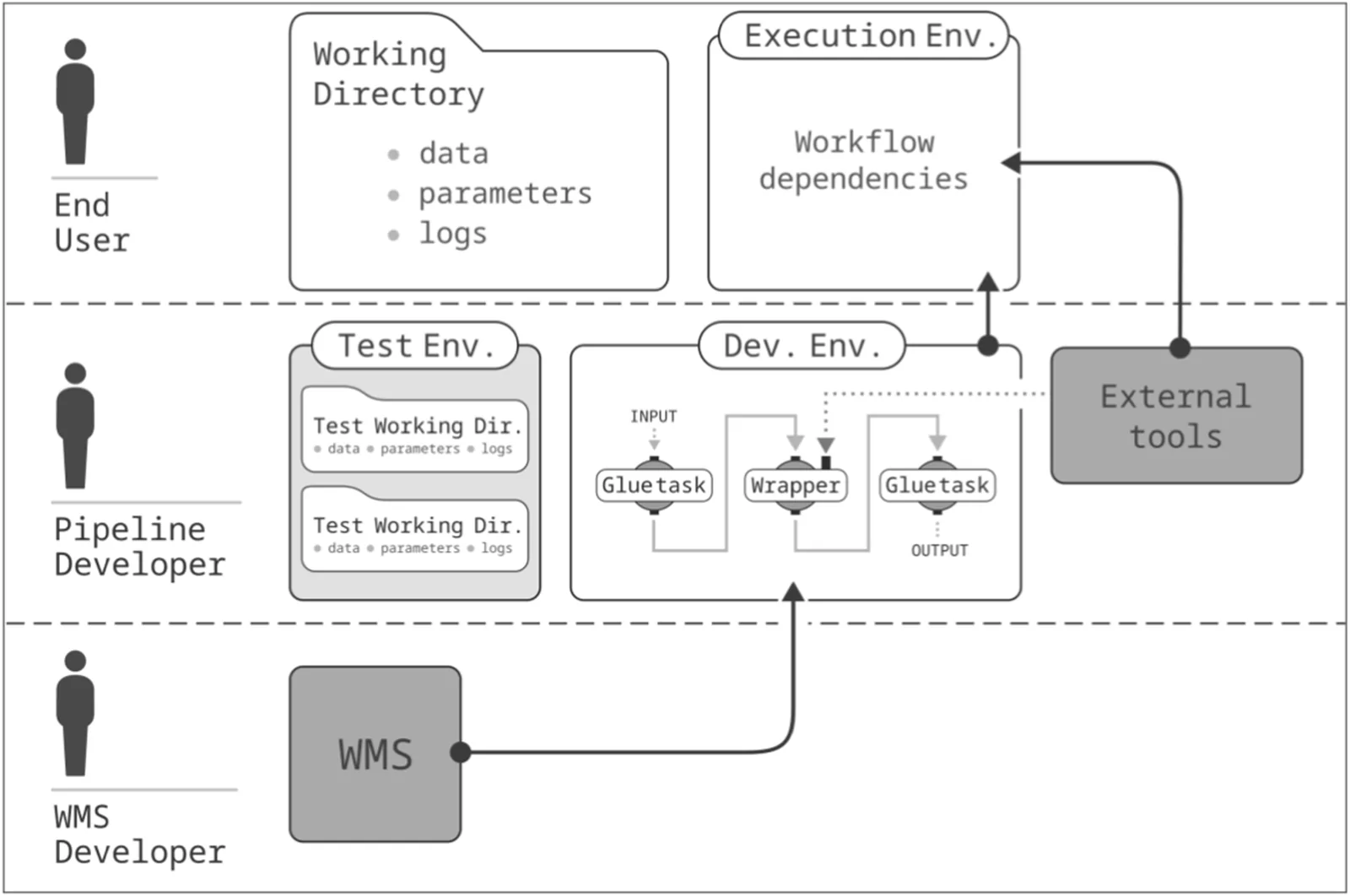
Pipeline development environment from WMS developers to end-users.

So far, we have revisited the SOLID principles and how they can be used to produce more flexible, reusable, and maintainable code, by elevating pipelines from “a collection of scripts” to engineered artefacts. We have also seen how the most common WMS support and promote the creation of modular pipelines made with reusable components, where the SOLID principles apply. While modular design and reusable libraries provide the foundation for maintainable pipelines, they must be complemented with practical measures that support robust execution in real-world usage. We would like to end this article with recommendations and good practices on three specific topics: robustness, project management and reproducibility. Figure 2 summarizes the environment in which pipelines are developed and used by end-users. We also comment on FAIR guidelines for research software.

### 4.1 Robustness

Well-designed modular components are necessary but not sufficient for robust pipeline execution. End users of a pipeline often have limited computing knowledge, which can lead to doubts about the pipeline’s usage, structure, limitations, and especially the correct preparation of input data. Even advanced users may struggle to debug some errors or failures, especially if they have not developed the pipeline. While most of these concerns should be addressed through detailed, user-oriented documentation, most errors remain common. One of the key aspects of ensuring analysis robustness is the implementation of checks before, during, and after the analysis is performed. The three most important types of checks are environment checks, input parameter checks, and result monitoring checks.

#### 4.1.1 Environment checks

While it is advisable for pipelines to be self-contained and not require manual installation of dependencies by the user, in practice there are usually prerequisites that must be satisfied by the system on which they run. Early verification of these requirements is essential to avoid silent failures or premature termination of the analysis, situations that are especially problematic in long-running executions. Therefore, it is advisable to perform systematic checks before starting execution. These checks should be isolated in atomic functions and ideally collected in libraries that facilitate their reuse. Our recommendation is to include common checks to deal with:

- Resources: Verification of the availability of necessary resources, such as the presence of a GPU when modules that use it exist, as well as compliance with minimum requirements for memory, storage, or computing capacity. This requires a prior study of the resources and resource scalability of the pipeline modules. It is especially important in pipelines containing massive alignment, assembly, or variant calling modules.
- Permissions: Verification that the process has read permissions on input data and read-write permissions on the output directory and temporary files. In pipelines intended to run exclusively on HPCs, it is also useful to verify that the analysis runs on a compute node.
- File system: Validation that the type of file system used is compatible with the analysis requirements, since certain implementations (e.g., FAT32 or exFAT) impose limitations on maximum file size, number of files, or supported metadata, which can affect the proper functioning of the pipeline. This is especially common in modules that generate large files such as SAMtools, BWA, or EDTA.
- Dependencies: Confirmation of the existence and accessibility of all external dependencies necessary for the correct execution of the pipeline. Even in dockerised pipelines, it is advisable to verify the existence of the container manager (e.g., Docker, Singularity, Podman).

#### 4.1.2 Input parameter checks

Preparing pipeline inputs is one of the most delicate and error-prone processes; therefore, it is necessary to dedicate special effort to adequately checking each and every input parameter. When configuration is partitioned per task, as the Interface Segregation Principle in Section 2 encourages, each task has a smaller and well-scoped set of parameters to validate, reducing both the risk of errors and the effort required to check them. Some of the most frequent checks that should be performed are:

- Type: Parameters provided by the user are of the correct type (numeric, text, boolean, etc.). For parameters that are paths to files or directories, it is also necessary to verify that those paths exist and that the files are in the expected format. In this latter case, the check should not be limited to the file extension but should also verify its content.
- Constraints: Pipelines often have constraints or incompatibilities between parameters. It is necessary to verify that these constraints are respected. Some common constraints are:
  ∘ Fixed values (IN): There are parameters that can only take a value from a fixed collection or range of values, for example,: True/False, 0/1, blastn/blastp/blastx, 1-100, etc.
  ∘ Alternatives (OR): These are constraints that apply to parameters that provide the same input in different formats. Consider, for example, the parameters “inputFasta” and “inputFastaDir”, where the first is the path to a FASTA file and the second is the path to a directory containing multiple FASTA files. If both parameters represent two ways of specifying the same input, their simultaneous use should generate an error or at least a warning.
  ∘ Limitations (NOT): These are constraints generally related to the *requested* system resources. This constraint is common in parameters used to allocate resources to a task. For example, requesting 10 parallel CPU tasks on a machine with only 5 CPUs, or selecting the “gpu” mode on a system without a GPU, must be rejected by the pipeline. Unlike environment checks, these checks ensure that the user’s resource request can be satisfied by the available hardware.

#### 4.1.3 Monitoring checks

This category includes all checks performed during the analysis process. The objective is to verify that the results are consistent with a successful analysis and to stop the process in time if they are not. In general, these checks are usually focused on detecting the presence or absence of results, understanding results as files and data. For example, a paralog detection task should skip the paralog removal task if no file is generated after alignment, or a contaminant detection task should warn the user if any is detected. Beyond standard task logs (input parameters, standard and error outputs), in pipelines processing large amounts of files, it is advisable to keep track of the number of input and output files each task receives and produces. This simple recommendation can help in debugging issues and also in re-executing parts of the analyses.

### 4.2 Standard project layout

This convention-over-configuration approach is well established in software development frameworks from other domains. For instance, Maven enforces a standard directory layout for Java projects, Rails follows the same principle for web applications, and frameworks like Next.js prescribe fixed project structures that teams adopt without discussion. Bioinformatics pipeline development benefits from the same discipline. A key aspect of project-level maintainability is consistency: when all pipelines in a team or organisation share the same internal structure, any developer can navigate an unfamiliar pipeline immediately, code libraries can be reused across projects without adaptation, and test environments can be automated against predictable paths. A practical way to enforce this consistency is to define a fixed layout, a standard directory and file structure, as a template stored in a version-controlled repository. Just as task interfaces in SOLID define contracts for data exchange, this project layout defines a contract for project organisation, enabling reliable assumptions about any pipeline built following it.

In this context, our proposal is to comply with a fixed project template. While implementation details may vary across projects and WMSs, our proposal is to stick to the same underlying principle: a clear separation between user-facing resources and the internal components required for pipeline execution. This separation promotes usability, facilitates maintenance, and reduces the risk of accidental modifications to operational files. Supplementary Material S1 provides a detailed description of project templates for Compi pipelines that can be easily adapted to other WMSs. As shown there, the public layout is intentionally minimal, exposing only the command-line interface, user documentation, and example parameter files. The private layout centralises workflow definitions, task scripts, environment-management utilities, and third-party dependencies under a single directory hierarchy.

In addition to the project template, a standard output organisation is recommended through the concept of a working directory (see panel C in Supplementary Material S1). Each analysis is executed in its own directory containing parameters, logs, and generated results in a predefined structure. By preserving the analysis inputs, execution records, and outputs within a single self-contained directory, this convention improves reproducibility, facilitates debugging, and enables automated processing of analysis outputs.

### 4.3 Reproducibility

From the developer’s perspective, reproducibility relies on the application of proper Software Configuration Management (SCM) practices. These include, on the one hand, the mandatory usage of a version control system (Git is the standard nowadays) to keep track of source code changes and versioning the pipeline. As mentioned earlier, it is also important to use containerization and environment locking (Docker/Conda/lockfiles), to provide reliable execution environments, both for pipeline development and for end-users, as discussed in Section 2.

With respect to end-users, the minimal reproducibility pack must take into account: software dependencies and versions, analysis parameters (settings) and input data. First, the exact version of the pipeline used to run every analysis must be recorded. This must be enough to be able to identify the versions of the pipeline dependencies used, and here it is important they are provided by Docker containers or Conda environments. At minimum, the working directory (see above) must include the exact parameters file (e.g., the compi.parameters in the case of Compi) and the exact pipeline version used for the analysis. It is also a good idea to include one or several scripts used to run the pipeline. Then, input data must be versioned to guarantee re-analysis of the same dataset (e.g., by dataset identifiers, checksums, or release dates), no matter if such data is placed within the working directory (for analysis-specific input files) or in a shared directory (e.g., genomes or big data files).

Finally, reproducibility must be supported by end-to-end tests that run the full pipeline using both real and simulated data, enabling validation of correctness and performance under controlled conditions. These tests are essential for pipeline developers and maintainers, and can even be automated (e.g., via continuous integration) so that regressions are detected whenever code, parameters, or dependencies change.

#### 4.3.1 Test environment

Designing a pipeline is as important as verifying that it produces the expected results. Building on the pipeline layout described above, it is straightforward to create a corresponding testing layout. A good testing layout should automate pipeline execution and its checks, provide access to outputs and logs, and allow rapid switching between different tests.

**FIGURE 3:**
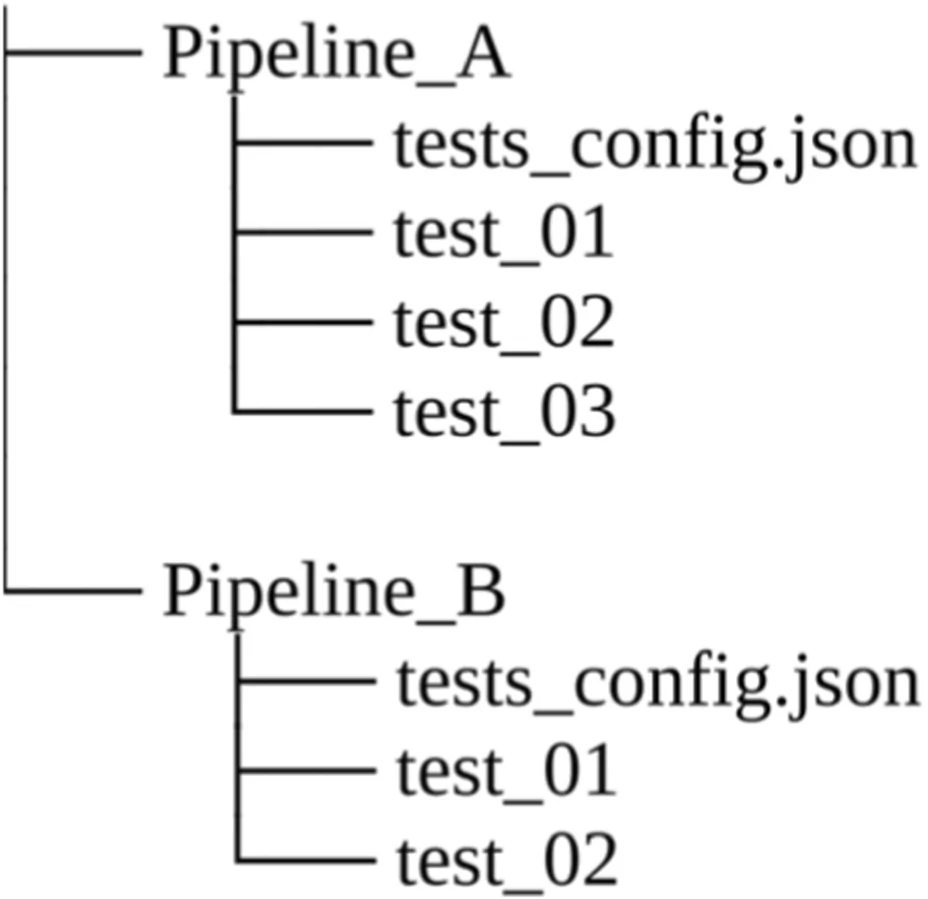
Example of test environment layout.

Because the pipeline layout (Figure 1 of Supplementary Material S1) specifies a directory to host the pipeline and the test script, automating tests and executions should be straightforward. Similarly, because the output layout (Figure 4, panel B) standardizes the results structure, accessing logs and environment variables is equally simple. Figure 3 shows an example structure for a testing environment.

Each pipeline has its own directory under which the different tests are grouped in subdirectories; each of them constitutes a working directory (Figure 4, panel B). The tests_config.json file stores metadata for each test: name, description, execution time, and timestamp of the last run. This information can be used by the test-management software to facilitate navigation between tests.

Beyond having an environment for executing tests, it is also important to have tools that enable the generation of synthetic data with known properties. The ability to anticipate results allows the design of tests to validate critical analysis points. Tools such as ART (Hua et al., 2012) for simulating Illumina sequencing by generating FASTQ files from FASTA; SeqModeller (Pérez-Rodríguez, 2026) for simulating mutations, insertions, and repetitions in FASTA genomes; or Seq-Gen (Rambaut and Grass, 1997) for generating sequences from evolutionary models and phylogenies, provide a wide variety of data for validating preprocessing, analysis, and quality-control modules.

Ideally, synthetic data design should follow the same principles of modularity, standardization, and reusability as the rest of the pipeline. It is useful to maintain a library of synthetic datasets that covers the most common assumptions of bioinformatics analyses. This includes generating sequencing data with varying quality scores, collections of sequences with different degrees of homology to a reference genome, and both contaminated and uncontaminated versions of the same sequences. Additionally, it is beneficial to have reduced versions of the test data that allow rapid execution of automated tests during development, without compromising the representativeness of the evaluated scenarios.

In any case, a pipeline should always ship with a minimal sample dataset and a reference set of expected outputs. This serves both as a user-facing example of the expected input conventions and parameters and as an executable regression test for installation, configuration, and workflow integrity.

### 4.4 FAIR guidelines for research software

Beyond the technical aspects commented previously, reproducibility, robustness, and long-term project maintainability are also supported by adherence to community guidelines for research software. Initiatives such as FAIR4RS (Barker et al., 2022) and FAIRsoft (Martín del et al., 2024) extend the FAIR principles to research software, providing recommendations to make pipelines and their components findable, accessible, interoperable, and reusable. These guidelines emphasize persistent identification and versioning, rich metadata and documentation, standardized interfaces, and clear licensing, all of which directly reinforce good software engineering practices and are in line with the specific recommendations presented in this work. Thus, aligning pipeline development as far as possible with FAIR4RS and FAIRsoft principles is also a good strategy to ensure that workflows remain usable, inspectable, and extensible over time, thereby improving reproducibility and reducing maintenance costs.

### 4.5 Further considerations

This work emphasizes reproducibility while also considering other software qualities such as robustness, maintainability, portability, and reusability. Reproducibility refers to the ability to rerun an analysis using the same code, parameters, data, and execution conditions and obtain consistent results. These other software qualities are closely related, but they are not equivalent, and while they do not by themselves guarantee reproducibility, they often establish the conditions that make reproducible analyses feasible, inspectable, and sustainable over time. Accordingly, the recommendations presented in the previous sections aim to support these qualities jointly, while keeping reproducibility as the central concern.

At the same time, it must be noted that reproducibility is also shaped by socio-technical factors that extend beyond pipeline architecture and implementation, including documentation practices, release governance, data stewardship, collaborative maintenance, and infrastructure evolution. The present work focuses more narrowly on engineering decisions within workflow systems and pipeline implementations, since these aspects are both directly actionable for developers and frequently under-specified in methodological guidance.

## 5 Case study

To illustrate how the principles discussed in this work can be applied in practice, we used the two-way-blast pipeline, originally developed a few years ago to identify orthologs through a reciprocal BLAST strategy (PEGI3S, 2026), as a refactoring case study. The goal was not only to improve the pipeline itself, but also to assess whether the practical vocabulary and engineering recommendations proposed here can guide an AI-assisted review and modernization process. To that end, we first created an end-to-end test to verify that the pipeline continued to produce the expected ortholog assignments after each change. We then prepared a dedicated Compi pipeline engineering skill, publicly available on GitHub (SING Group, 2026), that encodes the recommendations derived from this work together with Compi-specific guidance, examples, and useful commands. Using that skill in OpenCode, with Claude Sonnet 4.6 as the underlying model, we asked the agent to analyse the pipeline, propose a prioritized improvement plan, and implement the selected changes under our supervision. As documented in Supplementary Material S2, the agent produced a detailed issue report and implemented most of the proposed actions, resulting in a substantially improved version of the pipeline.
