# Supplementary material for: Software engineering for reproducible pipeline development in bioinformatics
Source: Front Bioinform. 2026 Sep 3;6:1824590. doi: 10.3389/fbinf.2026.1824590 (PMC13582464; doi:10.3389/fbinf.2026.1824590)
Supplement: Supplementary file 1 [file DataSheet1.pdf]

Supplementary Material S1

Software Engineering for Reproducible Pipeline Development in Bioinformatics

Daniel Pérez-Rodríguez[1], Alba Nogueira-Rodríguez[4], Jorge Vieira[2,3], Cristina P. Vieira[2,3], Daniel Glez-Peña[4], Hugo López-Fernández[4*]

[1]AA1 Research Group, Universidade de Vigo, Departamento de Química Física, Facultade de Ciencias, 32004 Ourense, Spain

[2]Instituto de Investigação e Inovação em Saúde (i3S), Universidade do Porto, Rua Alfredo Allen 208, 4200-135 Porto, Portugal

[3]Instituto de Biologia Molecular e Celular (IBMC), Rua Alfredo Allen, 208, 4200-135 Porto, Portugal

[4]SING Research Group, Galicia Sur Health Research Institute (IIS Galicia Sur), SERGAS-UVIGO, 36213 Vigo, Spain

***Correspondence:** Hugo López-Fernández; hlfernandez@uvigo.gal

Keywords: Bioinformatics, Software Engineering, Pipeline Development, Reproducibility

Supplementary Material 1

Figure 1 illustrates the proposed project template (panel A) alongside a representative layout commonly found in mature Compi pipelines generated via `compi-dk` (panel B). It is also included a working directory proposal (panel C).

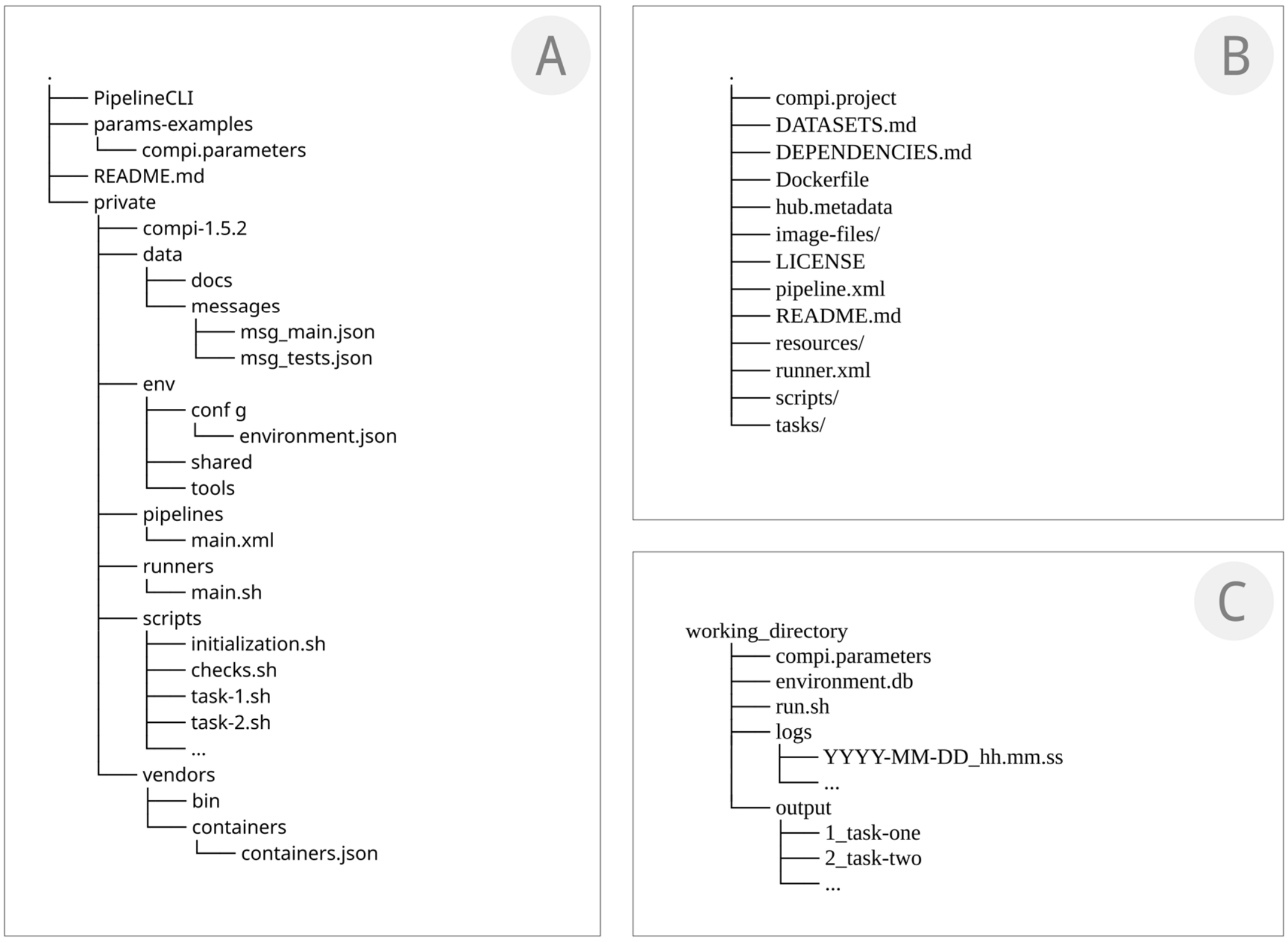

Figure 1: Panel A. Proposed standard project layout for bioinformatics pipelines following the software-engineering practices described in this work. Panel B. Representative layout of a mature Compi pipeline following the proposed software-engineering practices. Panel C. Example of an output layout based on the working-directory concept.

S1.1 Public layout

This is the portion of the pipeline intended for user interaction. It comprises the pipeline CLI, the `README.md` documentation, and the `params-examples` directory, which contains a complete example of a pipeline parameter file. The pipeline root directory is the user's first point of contact with the software, so it should be intentionally designed to be self-explanatory. Figure 4 of the main manuscript shows how a private directory has been allocated for supplementary documentation (accessible via links in `README.md`), and the examples have been grouped into a separate directory, reducing the root directory to just two files.

S1.2 Private layout

This part contains the files related to pipeline operation that are not intended for the end user. The private layout is centralized in the `private` directory:

- `private/data`: Supplementary documentation to `README.md` and pipeline messages, grouped in JSON files.

- `private/env`: Environment-management scripts organized into three subdirectories:
  - `config`: Contains a JSON file with all instructions passed to the environment manager at analysis time, including internal pipeline paths and result paths.
  - `shared`: Contains reusable code libraries; for example, functions for environment checks and user-parameter checks.
  - `tools`: Utilities used for environment management, container handling, and messaging. Accessible from any task.
- `private/pipelines`: Pipeline definition files in XML, required by Compi to chain tasks and manage task flow. Contains the main workflow XML (`main.xml`).

- `private/runners`: Private scripts for analysis initialization. The user interacts with these runners through the CLI. Includes the runner prepared to execute the main pipeline (`main.xml`).

- `private/scripts`: Scripts defining the tasks that compose the pipeline; this is where most of the development work for a new pipeline occurs. Includes `initialization.sh`, which prepares the environment based on `environment.json` and `containers.json` (see next point), and `checks.sh`, where environment and parameter tests are added.

- `private/vendors`: External dependencies of the pipeline. Contains the `bin` subdirectory for binaries and `containers`, with the `containers.json` file defining the containers used in the analysis. This file is processed by the container-management tool, which downloads and tests containers at the start of the analysis.

S1.3 Working directory

In addition to public and private layouts, it is necessary to define an output layout—namely, a convention for the structure of pipeline results that is independent of the type and number of outputs the pipeline may produce. The concept of a “working directory” is helpful here: encourage the use of one directory per analysis, where parameters, logs, and results are colocated in a predefined structure.

Panel C on figure 4 (main manuscript) shows an example of an output layout based on the working-directory concept. The analysis parameters and the SQLite database containing the analysis environment variables reside in the root directory, while `logs` and `output` are placed in two subdirectories. The `logs` directory stores records of different runs, and `output` contains one subdirectory per pipeline task.
