# Supplementary material for: Software engineering for reproducible pipeline development in bioinformatics
Source: Front Bioinform. 2026 Sep 3;6:1824590. doi: 10.3389/fbinf.2026.1824590 (PMC13582464; doi:10.3389/fbinf.2026.1824590)
Supplement: Supplementary file 2 [file DataSheet2.pdf]

Supplementary Material S2

Software Engineering for Reproducible Pipeline Development in Bioinformatics

Daniel Pérez-Rodríguez[1], Alba Nogueira-Rodríguez[4], Jorge Vieira[2,3], Cristina P. Vieira[2,3], Daniel Glez-Peña[4], Hugo López-Fernández[4*]

[1]AA1 Research Group, Universidade de Vigo, Departamento de Química Física, Facultade de Ciencias, 32004 Ourense, Spain

[2]Instituto de Investigação e Inovação em Saúde (i3S), Universidade do Porto, Rua Alfredo Allen 208, 4200-135 Porto, Portugal

[3]Instituto de Biologia Molecular e Celular (IBMC), Rua Alfredo Allen, 208, 4200-135 Porto, Portugal

[4]SING Research Group, Galicia Sur Health Research Institute (IIS Galicia Sur), SERGAS-UVIGO, 36213 Vigo, Spain

***Correspondence:** Hugo López-Fernández; hlfernandez@uvigo.gal

Keywords: Bioinformatics, Software Engineering, Pipeline Development, Reproducibility

Supplementary Material 2

This supplementary section documents the interaction used to review and refactor the `two-way-blast` pipeline (https://github.com/pegi3s/two-way-blast) through an AI-assisted workflow. Before starting the refactoring process, we created an end-to-end test based on the existing sample dataset. This test was used throughout the process to verify that the pipeline continued to identify the same orthologs after each change.

Next, we installed in OpenCode the Compi pipeline engineering skill. All interactions described below were carried out in OpenCode using Claude Sonnet 4.6 as the underlying model.

The process started with an analysis step. The initial prompt, issued in **plan mode**, was the following:

> Analyze this Compi pipeline.

After obtaining the report, we switched to **build mode** and issued the following prompt:

> Write down this report into ANALYSIS.md, and include, at the end, a new implementation plan for all items, ranked by priority.

The resulting `ANALYSIS.md` file included, among other details, the following table summarizing the main findings and the corresponding recommendations:

File	Issue	Principle / Recommendation	Suggestion
`pipeline.xml`	All 5 tasks (`initialization`, `prepare-files-and-dbs`, `split-reference`, `create-combinations-file`, `run-blast`) have inline code — any change to logic requires editing the pipeline XML directly	OCP	Extract each task body to `tasks/<task-id>.sh` and add `src="tasks/<task-id>.sh"` to each task element
`pipeline.xml`	`prepare-files-and-dbs` has two distinct responsibilities: (1) FASTA linearization/normalization via multiple `sed`	SRP	Split into two tasks: `normalize-fastas` (extracts the `sed` chain) and `create-blast-dbs` (runs `makeblastdb`); connect them with an `after="*normalize-fastas"` iteration-level dependency

File	Issue	Principle / Recommendation	Suggestion
	steps, and (2) `makeblastdb` database creation		
`pipeline.xml`	`blast_type` is listed in `params="blast_type"` of `prepare-files-and-dbs` but is never referenced in that task’s body (`-dbtype nucl` is hardcoded)	ISP	Remove `blast_type` from the `params=` attribute of `prepare-files-and-dbs`
`pipeline.xml`	`blast_type` accepts any free-form string with no validation — invalid values (e.g. `tblastn`) will silently produce empty or incorrect results	Robustness — input checks	Add an IN constraint in `initialization`: check that `blast_type` is one of `blastn`, `tblastx`; exit with a descriptive error message otherwise

File	Issue	Principle / Recommendation	Suggestion
`pipeline.xml`	No check that `output_dir` exists or is writable before tasks begin	Robustness — input checks	Add `mkdir -p "${output_dir}"` and a write-permission test in `initialization`
`pipeline.xml`	No check that `input_dir` contains at least one file	Robustness — input checks	Add a guard in `initialization`: if `input_dir` is empty or contains no regular files, abort with a clear message
`pipeline.xml`	Tool availability of `makeblastdb`, `seqkit`, and the `blast_type` binary (e.g. `tblastx`) is never verified before use	Robustness — env checks	Add `command -v makeblastdb`, `command -v seqkit`, and `command -v "${blast_type}"` checks in `initialization`
`pipeline.xml`	`prepare-files-and-dbs` and `create-combinations-file` enumerate files with `ls ${input_`	Robustness — monitoring	Replace `ls`-based file enumeration with `find ... -maxdepth 1 -type f -printf '%f\n'` or equivalent

File	Issue	Principle / Recommendation	Suggestion
	`dir}/*` and `ls ${working_dir}/...` — this breaks silently on filenames containing spaces		
`pipeline.xml`	`run-blast` uses unquoted `$seqID1` and `$seqref` in `grep -w` — FASTA sequence IDs with special characters (parentheses, pipes, dots) can cause `grep` to mismatch or produce an error	Robustness — monitoring	Quote variables and use `grep -wF "$seqID1"` (fixed-string mode) to prevent regex interpretation
`pipeline.xml`	No file-count tracking in the	Robustness — monitoring	Log the number of combinations processed and output files

File	Issue	Principle / Recommendation	Suggestion
	`run-blast` foreach: a silent BLAST failure for one combination leaves no trace in logs		written; optionally add a post-`run-blast` monitoring task that checks expected vs. actual `.orthologs` files
`Dockerfile`	Base image `FROM pegi3s/seqkit` has no version tag — resolves to `latest`, which is a mutable moving target	Reproducibility	Pin to an explicit tag, e.g. `FROM pegi3s/seqkit:0.11.0` (consistent with `DEPENDENCIES.md`)
`tests/two-way-blast-1/run.sh`	`rm -rf "${OUTPUT_DIR}/*"` — the glob is inside double quotes and does **not** expand; the literal string `${OUTPUT_DIR}/*` is passed	Test infrastructure	Use `rm -rf "${OUTPUT_DIR:?}/"*` (glob outside the quotes) to correctly remove all files in the output directory

File	Issue	Principle / Recommendation	Suggestion
	to `rm -rf`, which attempts to remove a file named literally `*`		
`tests/two-way-blast-1/download.sh`	Test data is downloaded from the `master` branch (`raw/master/resources/...`) — a moving target that can change without notice and break test reproducibility	Reproducibility	Pin the download URL to a specific release tag or commit SHA

We then continued in build mode and introduced the following prompts, with no additional intervention between interactions:

> Let’s tackle P2.1 first as it is needed to build the image properly. Do it, verify building and testing. If succeed, do a commit.
>
> Let’s tackle P1.1 and P1.2.
>
> Now let’s tackle all priority 4 items (P4.1, P4.2, P4.3, P4.4 and P4.5) in a new commit. After that is done and tested, tackle P5.1 in a separate commit.
>
> Now let’s tackle P7.1 and P3.1, in separate commits.
>
> Now let’s tackle P3.2, P3.3 and P3.4, in the same commit.

The second prompt produced a single commit, not two as one might expect, so the following interactions stated the desired commit grouping more explicitly. During implementation, the agent verified pipeline correctness with `compi validate` and ran the end-to-end test through the provided script. The resulting sequence of commits was:

```
0b462f1 Add tool availability, input and output directory checks (P3.2, P3.3, P3.4)
a8fd0c4 Validate blast_type value in initialization (P3.1)
bad1df3 Replace ls-based file enumeration with find (P7.1)
d28223d Split prepare-files-and-dbs into normalize-fastas + create-blast-dbs (SRP, P5.1)
58da8fb Extract all inline task code to external scripts (OCP, P4.1-P4.5)
9651db1 Fix correctness bugs in grep calls and test output cleanup
39de1a1 Fixes README
4458663 Pin base image to pegi3s/seqkit:0.11.0 for reproducibility
```

The agent also failed to update the pipeline version in `pipeline.xml`, so that change was made manually afterwards. To conclude the exercise, we switched back to **plan mode** and asked for a summary:

> Check the implementation plan and the latest commits and produce an executive summary of what has been done and what still needs to be done.

That summary was then saved into a file by issuing the following prompt in **build mode**:

> Write down this into ANALYSIS_SUMMARY.md

The resulting summary included the following table:

Item	Description	Commit
**P2.1**	Pin `FROM pegi3s/seqkit:0.11.0` in Dockerfile	`4458663`
**P1.1**	Fix `rm -rf` quoting bug in test runner	`9651db1`
**P1.2**	Fix unquoted `$seqID1` in `grep` calls → `grep -wF`	`9651db1`
**P4.1–P4.5**	Extract all 5 inline task bodies to `tasks/*.sh`; add `src=` to each task; update Dockerfile	`58da8fb`
**P5.1**	Split `prepare-files-and-dbs` → `normalize-fastas` + `create-blast-dbs`	`d28223d`
**P6.1**	Remove spurious `params="blast_type"` (resolved as side-effect of P5.1)	`d28223d`
**P7.1**	Replace `ls`-based enumeration with	`bad1df3`

Item	Description	Commit
	`find` in `pipeline.xml` and `create_combinations_file.sh`	
**P3.1**	Validate `blast_type` IN constraint in `initialization`	`a8fd0c4`
**P3.2–P3.4**	Tool availability check, non-empty `input_dir` check, writable `output_dir` check	`0b462f1`

It also identified the following residual issues discovered during implementation:

Finding	Detail
**Orphaned script**	`tasks/prepare_files_and_dbs.sh` is still in the repo and committed, but is no longer referenced by any task after P5.1 split it into `normalize_fastas.sh` and `create_blast_dbs.sh`. It should be deleted.
**Compi version constraint**	The `*` iteration-level dependency syntax (e.g. `after="*normalize-fastas"`) is not supported by the bundled `compi 1.2.3`. Plain `after="normalize-fastas"` was used instead, which is correct but coarser. Upgrading `compi.project` to a newer Compi version would unlock this.
**`ANALYSIS.md` uncommitted**	The analysis document is untracked and has never been committed.
